# Fluctuating hearing loss associated with a novel heterozygous variant in the TNC gene related to nonsyndromic hearing loss

**DOI:** 10.1016/j.bjorl.2025.101577

**Published:** 2025-04-08

**Authors:** Mariana Carolina Castellanos-Acevedo, Juan C. Ospina-García, Carlos Alberto Restrepo-Chamorro

**Affiliations:** a Pontifical Xaverian University, School of Medicine, San Ignacio University Hospital, Bogotá, Colombia; b Otolaryngology Unit, San Ignacio University Hospital, Bogotá, Colombia

## Introduction

Hearing loss is a significant public health issue worldwide, affecting over 5% of the global population, which translates to approximately 1.5 billion individuals. By 2050, this figure is expected to increase to 2.5 billion.^1^

Congenital hearing loss affects about 1 to 3 per 1,000 live births. External factors account for approximately half of these cases, while genetic alterations contribute to the other half. Key genes implicated include SLC26A4, MT-RNR1, and GJB2, with the latter responsible for up to 50% of prelingual hearing impairments in the Caucasian population.^2^

The TNC gene (OMIM *187380) encodes an extracellular matrix glycoprotein called Tenascin-C, expressed during embryonic morphogenesis and in certain adult locations such as the inner ear, specifically in the spiral lamina and basilar membrane, where it is a major component along with collagen IV and laminin. Mutations in any component of the basilar membrane could lead to ionic imbalance between perilymph and endolymph, resulting in hearing loss.^3^

The following presents the case of a patient with not known family history of hearing impairment, experiencing fluctuating postlingual hearing loss with a mutation in the TNF gene, previously described only twice but with a new variant of uncertain significance not reported in databases. A brief review of the information regarding the mutation in this gene will be conducted.

## Clinical case presentation

The article presents a clinical case of a 9-year-old male patient diagnosed with fluctuating bilateral hearing loss associated with intermittent non-pulsatile tinnitus. The hearing loss was first detected around four years prior. The patient has no notable prenatal history, was born at full term, and only experienced neonatal jaundice, which was treated effectively with phototherapy. During preschool years, he had a history of otitis media on four occasions but no other significant medical history. Importantly, there is no family history of hearing loss or genetic syndromes.

**Fig. 1:**
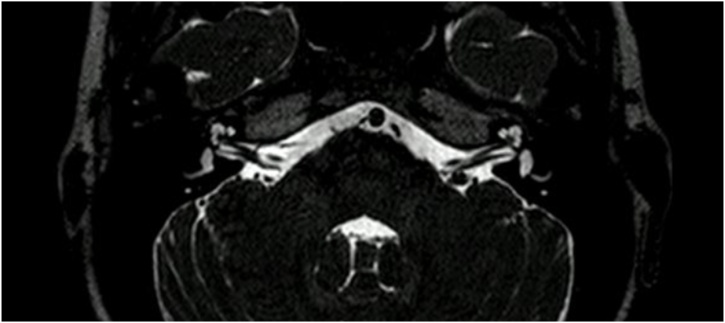
Imaging study.

**Fig. 2:**
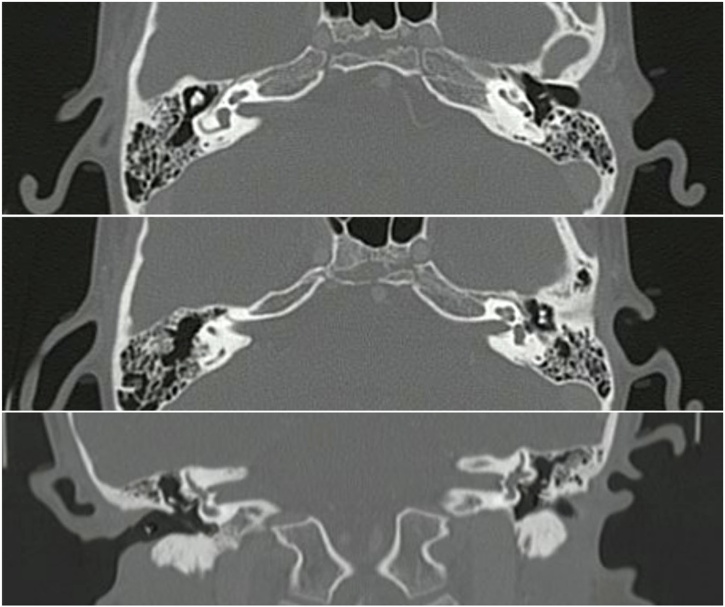
Imaging study.

Initial evaluations included physical examinations and audiometric studies. Otoscopy revealed no abnormalities, while Rinne’s and Weber’s test indicating normal function. The patient exhibited appropriate language acquisition and psychomotor development. Imaging studies, including a contrasted ear tomography and brain MRI, were performed, revealing only vascular loops in proximity to the VII‒VIII complexes. A neurosurgical evaluation ruled out these findings as the cause of his symptoms (Fig. 1, Fig. 2).

Audiometric evaluations began in 2019, revealing preserved auditory sensitivity in the left ear and mild high-frequency hearing loss in the right ear, with normal discrimination at 30 dB. However, subsequent audiometries indicated a progression to bilateral moderate conductive hearing loss. In November 2020, the patient underwent adenoidectomy and tympanostomy tube placement, but the mother reported no improvement in hearing.

**Fig. 3:**
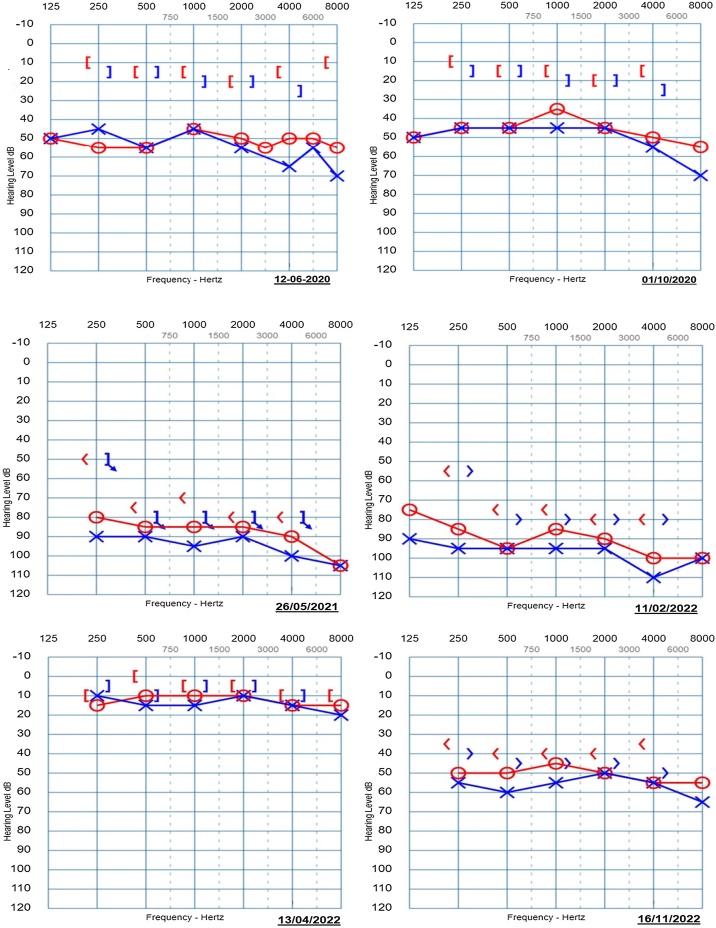
Audiometric evaluations.

Further audiometric assessments demonstrated inconsistent responses. By May 2021, profound bilateral sensorineural hearing loss was recorded, with discrimination percentages dropping significantly. Notably, in 2022, the mother observed fluctuating improvements in hearing, with some days showing better auditory sensitivity. Audiometric evaluations in February indicated severe to profound bilateral sensorineural hearing loss, but by April, results showed preserved auditory sensitivity with high discrimination rates. However, November results again indicated moderate bilateral sensrineural hearing loss (Fig. 3).

Given these findings, a genetic evaluation was initiated, leading to the identification of a heterozygous variant of uncertain significance in the TNC gene through Next-Generation Sequencing (NGS). This variant involved a substitution of cytosine with thymine at position 1615 of the cDNA (c.1615C > T) in exon 3 of the gene, potentially introducing a premature stop codon. However, the variant’s pathogenicity remained ambiguous, as it has not been reported in existing databases.

## Discussion on tnc gene and hearing loss

Since 1999, a certain role of tenascin in the development of the murine cochlea has been reported. Extracellular matrix components have been shown to influence the growth and adhesion of spiral ganglion neurons and cochlear hair cells during mouse development. Moreover, tenascin-C has an inhibitory and anti-adhesive role in the postnatal mouse inner ear, acting as a boundary-forming molecule, influencing the growth of spiral ganglion fibers.^4^

In 2013, mutations in the TNC gene, which encodes tenascin-C, were first described as causing nonsyndromic hearing loss. This involved a five-generation family in China with autosomal dominant hereditary hearing loss located on chromosome 9q31.3-34.4, where a new heterozygous mutation in exon 19 (c.5317G > A, p.V1773M) was identified using a combined linkage analysis and whole-exome sequencing method.^3^

In 2019, Jin X et al. conducted a genetic variant analysis in 92 Chinese patients with nonsyndromic hearing loss, where they identified a single patient (sporadic case) with a mutation in the TNC gene (c.1641C > A, p.C547X). However, no clinical characteristics of this patient were described.^2^ Recently, a novel splice-altering mutation (c.5247A > T, p.Gly1749Gly) in the TNC gene was identified as responsible for autosomal dominant non-syndromic hearing loss in a five-generation Chinese family.^5^

In our patient, a novel heterozygous variant in the TNC gene was identified, involving the substitution of cytosine with thymine at position 1615 of the cDNA (c.1615C > T) in exon 3 of the gene. At the protein level, this variant potentially introduces a premature stop codon at amino acid 539 in a 2,201-amino acid protein (p.Gln539*). While this variant likely results in an mRNA transcript degraded by the Nonsense-Mediated Decay (NMD) system, leading to a deleterious effect on the protein, truncated variants are not a known mechanism of pathogenicity for this gene.

While this variant likely produces an mRNA transcript degraded by the Nonsense-Mediated Decay (NMD) system, leading to a deleterious effect on the protein, truncated variants are not a known mechanism of pathogenicity in this gene. A review of databases found no reports of this variant in ClinVar, The Human Gene Mutation Database (HGMD), Leiden Open Variation Database (LOVD), or the consulted scientific literature. Its allelic frequency is very low (< 0.0001, gnomAD), and it has been described in healthy individuals.

Considering the lack of information on the variant's implication and following the American College of Medical Genetics and Genomics (ACMG) guidelines, this variant is classified as a Variant of Uncertain Significance (VUS) and meets the classification criteria PP3 (computational predictors classify the variant as deleterious) and BS2 (reported in healthy individuals).

## Conclusion

In our study, a novel heterozygous variant in the TNC gene was identified, resulting in the substitution of cytosine with thymine at position 1615 of the cDNA (c.1615C > T) in exon 3 of the gene responsible for bilateral fluctuating sensorineural hearing loss. This is the first case reported in the literature of this variant of uncertain significance.

## Funding

This research received no specific grant from any funding agency in the public, commercial, or not-for-profit sectors.

## Declaration of competing interest

The authors declare no conflicts of interest.
